# A Variable Kinematic Multifield Model for the Lamb Wave Propagation Analysis in Smart Panels

**DOI:** 10.3390/s22166168

**Published:** 2022-08-17

**Authors:** Jamal Najd, Enrico Zappino, Erasmo Carrera, Walid Harizi, Zoheir Aboura

**Affiliations:** 1 Mul2 Group, Department of Mechanical and Aerospace Engineering, Politecnico di Torino, Corso Duca degli Abruzzi 24, 10129 Torino, Italy; 2 Centre de Recherche Royallieu, Roberval (Mechanics Energy and Electricity), Université de Technologie de Compiègne, CEDEX CS 60 319, 60 203 Compiègne, France

**Keywords:** plate models, Carrera Unified Formulation, Lamb wave, piezo-patch, node-dependent kinematics

## Abstract

The present paper assessed the use of variable kinematic two-dimensional elements in the dynamic analysis of Lamb waves propagation in an isotropic plate with piezo-patches. The multi-field finite element model used in this work was based on the Carrera Unified Formulation which offers a versatile application enabling the model to apply the desired order theory. The used variable kinematic model allowed for the kinematic model to vary in space, thereby providing the possibility to implement a classical plate model in collaboration with a refined kinematic model in selected areas where higher order kinematics are needed. The propagation of the symmetric ($S_{0}$) and the antisymmetric ($A_{0}$) fundamental lamb waves in an isotropic strip was considered in both mechanical and piezo-elastic plate models. The convergence of the models was discussed for different kinematics approaches, under different mesh refinement, and under different time steps. The results were compared to the exact solution proposed in the literature in order to assess and further determine the effects of the different parameters used when dynamically modeling a Lamb wave propagating in such material. It was shown that the higher order kinematic models delivered a higher accuracy of the propagating wave evaluated using the corresponding Time Of Flight (TOF). Upon using the appropriate mesh refinement of 2000 elements and sufficient time steps of 4000 steps, the error between the TOF obtained analytically and numerically using a high order kinematics was found to be less than 1% for both types of fundamental Lamb waves $S_{0}$ and $A_{0}$. Node-dependent kinematics models were also exploited in wave propagation to decrease the computational cost and to study their effect on the accuracy of the obtained results. The obtained results show, in both the mechanical and the piezo-electric models, that a reduction in the computational cost of up to 50% can be easily attained using such models while maintaining an error inferior to 1%.

## 1. Introduction

Unlike Rayleigh waves, or surface acoustic waves that propagate close to the free surface of a structure with a penetration of the order of the wavelength, guided waves are a type of elastic waves that propagate in a thin plate or shell-like structure and remain confined within the boundaries of that structure—hence the term guided. The propagation of ultrasonic guided waves was first described in the work of the mathematician Horace Lamb [1], after which [2,3] published an extensive theory on such waves. This type of wave is characterized by its ability to travel over large distances with little energy loss, highlighting their desirability and suitability to be used in non-destructive health monitoring applications, specifically in the ultrasonic inspection of structures. Guided waves are classified into two distinguishable types, the shear horizontal (SH) type, and the Lamb waves which are also referred to as guided plate waves as they are guided by the free parallel upper and lower surfaces of the plate.

**Figure 1:**
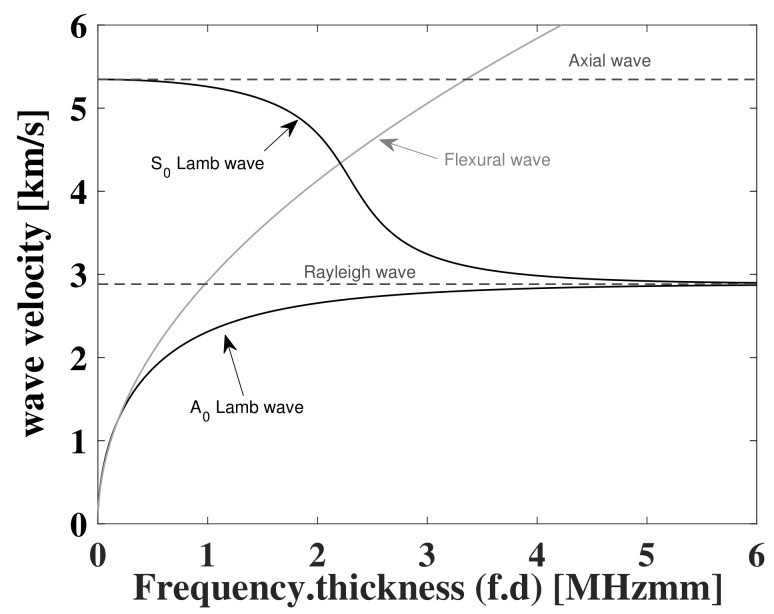
Frequency dependence of wave velocities for axial, flexural, Lamb, and Rayleigh waves in 1-mm thick aluminium plate.

The difference between the two is in the particle motion of the propagating wave. The SH are horizontally polarized, and the shear-particle motion is contained within the horizontal plane (parallel to the plate surface) perpendicular to the direction of wave propagation, whereas Lamb waves are vertically polarized, and the particle motion is elliptical, contained in the plane determined by the direction of the propagating waves and the normal direction of the plate [4]. Both types of waves can be classified into symmetric and antisymmetric waves, and except for the fundamental symmetric mode of SH waves, all other wave modes are of a dispersive nature, meaning that the group velocity depends on the frequency of the propagating wave. In SH waves, the fundamental symmetric mode can be found at all frequencies, and every other mode has a critical frequency $W_{cr}$ at which it appears together with the previous modes. In Lamb waves on the other hand, and for a low frequency-thickness f.d coefficient, both fundamental modes $S_{0}$ and $A_{0}$ can be seen. As this coefficient increases, other modes start appearing and propagating simultaneously, depending on the dispersion curves of the material at hand. As f.d→∞, the symmetric and antisymmetric modes degenerate into Rayleigh waves, as the thickness becomes comparable and greater than a wavelength, see Figure 1. On the other hand, as f.d→0, the $S_{0}$ mode vanishes to an axial or longitudinal wave, and the $A_{0}$ mode vanishes to a plate flexural wave [4]. It is of interest to carry out the study at low frequencies, due to the lower dispersion of $S_{0}$ and $A_{0}$ modes at such frequencies and the lower complexity of the problem due the ability to excite a specific mode [5].

In addition to the characteristics of the guided waves above, and due to their transmission, reflection, scattering and absorption, they are used in damage detection in the structures. Guided waves, and especially Lamb waves, were used to identify damages of metallic structures, such as fatigue crack and corrosion, damage orientation and to estimate the remaining useful life (RUL) [6,7,8]. A comprehensive review about the use of guided waves in structural health monitoring (SHM) and damage detection of metallic structures was performed [9]. The detection and identification of the different damages in composite materials, fiber and matrix cracking, delamination and voids, through the use of these waves was also studied in the literature despite the attenuation of these waves in fiber reinforced composites. Using Lamb waves, the real-time delamination damage detection of different damage severities was experimentally studied by [10], accompanied by a numerical FEM model to detect different localized damages. Fatigue induced delamination, transverse ply crack and hole damages were studies by [11], whereas turning modes due to delamination were experimentally and numerically studied using $A_{0}$ Lamb mode in [12]. A novel ultrasonic-based Lamb wave technique was proposed by [13], which could provide more information, being more sensitive to the local effects of damage in a material. Damage localization and identification using circular defects were experimentally and numerically studied by [14], implementing two Matlab algorithms under different fiber orientations. On the other hand, hole damage localization was experimentally studied in [15], adopting the power spectral density method and Lamb wave tomography which could be used under a high noise environment. A review on the identification of damage in composite structures using guided waves was established by [16]. The complexity of using Lamb waves lies in the dispersive nature, multi-modal excitation and mode conversion which make it difficult to analyse the signals and to identify the damage [17]; thus, it is essential to specify a frequency which identifies the problem requirements, around which the narrowband excitation can be implemented, favouring the use of a windowed tone burst over pulse excitation.

Guided Waves can be excited using different means, which are grouped into five different categories of sensors/actuators [16], namely an ultrasonic probe, laser, piezoelectric element, interdigital transducer (PVDF and other novel PVDF-like transducers), and optical fiber. The use of piezoelectric transducers is most widely implemented due to the wide range of advantages delivered, from the high performance, suitability for embedding and integration, negligible mass and dimensions, high mechanical strength with wide frequency response and their low price, to name a few. These transducers can be used to excite symmetric and antisymmetric Lamb waves, when their polarization is along the direction of the applied electric field, denoted by $d_{13}$ or $d_{33}$ transducers. They can also be used to excite shear horizontal waves (SH) if they are polarized in a perpendicular direction to the applied electric field signal, denoted by $d_{15}$ transducers. The use of $d_{13}$ and $d_{33}$ transducers has been widely researched, however, recent studied have focused on using shear transducers due to the higher coupling coefficients in shear excitation, and due to the mono-excitation direction [18,19]. On the other hand, Ref. [20] studied the antisymmetric Lamb wave excitation of a shear-deforming PZT embedded at the neutral axis. They also carried out a parametric study to evaluate the effects of offsetting the actuator from the neutral axis, which resulted in the actuation of both antisymmetric and symmetric waves. Ref. [21] proposed a new $d_{36}$ wafer type, and studied the generated guided waves in comparison with the traditional $d_{13}$ actuator.

The numerical modelling of propagating Lamb waves has been demonstrated in the literature by many studies [22,23,24,25,26], in which commercial FEMs were extensively used (i.e., Ansys, Abaqus, Comsol, etc.) as a verification of the experimental results. The models obtained have a high computational cost as it is essential to use a 3-D solid element to model the transducers and the structures in order to obtain a good model. It is possible to reduce the computational cost by adopting the effective material properties in a laminated structure, or by adopting a shell model, to acquire displacements, and neglecting the inter-laminar stresses [27]. For example, Abaqus uses 3-D shell (S4R) elements by applying the Mindlin–Reissner plate theory. This allows for the rotation of the normal to the plate, favouring the non- orthogonality of these normal lines to the mid-plane after deformation, allowing for transverse shear deformation effects. In [28], the attenuation of lamb waves in composite materials was modelled by introducing a damping coefficient, specifically Rayleigh damping.

In order to better describe the propagating waves, higher order beam and plate theories were implemented in transient analysis [29,30,31,32]. A comparison between the different higher order models was provided by [33], where the accuracy of each model was assessed according to different discretization schemes. The benchmark proposed in their study was adopted in the current work, where a similar but extensive comparison was carried out, using plate models with different kinematics through-the-thickness, comparing the effect of mesh density, time step, model kinematics and plate element type. The used numerical model is the in-house MUL2 FE implicit dynamic model, based on the Carrera unified formulation (CUF). An aluminium strip was modelled, and unidirectional wave propagation along the length of the strip was carried out. Both mechanical and electromechanical models were used as the propagating wave was excited in two different ways, mechanically applying nodal forces and electrically applying an electric potential. The novelty of this work, besides the extensive convergence analyses of the 2-D mechanical and the proposed electro-mechanical models is that a Node-dependent Kinematics approach was considered to study the wave propagation using reduced local kinematics models with a reduced computational time.

## 2. Refined Two-Dimensional Electro-Mechanical Model

### 2.1. Preliminaries

The general 4×1 generalized unknowns vector q for an electro-mechanical model, taking the electric potential ϕ as a primary variable, can be written as follows:

(1) $$q={\left\{u_{x},u_{y},u_{z},\phi \right\}}^{T}$$

The 3×1 electric field vector E can be derived as follows:

(2) $$E={\left\{E_{x},E_{y},E_{z}\right\}}^{T}={\left\{\partial_{x},\partial_{y},\partial_{z}\right\}}^{T}\phi$$

The generalized 9×1 strain vector, $\bar{\varepsilon }$, can be written as follows:

(3) $$\bar{\varepsilon }={\left\{\varepsilon_{xx},\varepsilon_{yy},\varepsilon_{zz},\varepsilon_{xz},\varepsilon_{yz},\varepsilon_{xy},E_{x},E_{y},E_{z}\right\}}^{T}=Dq$$

where the matrix of the differential operator D is a 9×4 matrix given by:

(4) $$D=\left[\begin{array}{cccc} \frac{\partial }{\partial x} & 0 & 0 & 0\\ 0 & \frac{\partial }{\partial y} & 0 & 0\\ 0 & 0 & \frac{\partial }{\partial z} & 0\\ \frac{\partial }{\partial z} & 0 & \frac{\partial }{\partial x} & 0\\ 0 & \frac{\partial }{\partial z} & \frac{\partial }{\partial y} & 0\\ \frac{\partial }{\partial y} & \frac{\partial }{\partial x} & 0 & 0\\ 0 & 0 & 0 & \frac{\partial }{\partial x}\\ 0 & 0 & 0 & \frac{\partial }{\partial y}\\ 0 & 0 & 0 & \frac{\partial }{\partial z} \end{array}\right]$$

The coupled constitutive equations (stress-charge form or *e-form*) can be expressed by:

(5) $$\begin{array}{cc} \sigma & =C^{E}\varepsilon -e^{T}E\\ D_{e} & =e\varepsilon +\chi^{S}E \end{array}$$

where σ is the 6 × 1 mechanical stress vector, C the 6 × 6 matrix of mechanical material coefficients, and ε is the 6 × 1 strain vector, expressed as follows:

(6) $$\varepsilon ={\left\{\varepsilon_{xx},\varepsilon_{yy},\varepsilon_{zz},\varepsilon_{xz},\varepsilon_{yz},\varepsilon_{xy}\right\}}^{T}=Du$$

with $D_{e}$ being the 3 × 1 electric displacement vector, χ the 3 × 3 permittivity matrix, and e the 3 × 6 piezoelectric coupling matrix. The superscripts *E* and *S* denote that the corresponding quantities are evaluated under constant electric field and strain, respectively, while the superscript *T* denotes the transpose of a matrix.

The general compact form of Equation (5) can be expressed as follows:

(7) $$\bar{\sigma }=\tilde{H}\bar{\varepsilon }$$

which explicitly can be expressed as in Equation (8)

(8) $$\left\{\begin{array}{c} \sigma_{xx}\\ \sigma_{yy}\\ \sigma_{zz}\\ \sigma_{xz}\\ \sigma_{yz}\\ \sigma_{xy}\\ D_{e_{x}}\\ D_{e_{y}}\\ D_{e_{z}} \end{array}\right\}=\left[\begin{array}{ccccccccc} C_{11}^{E} & C_{12}^{E} & C_{13}^{E} & 0 & 0 & C_{16}^{E} & -e_{11} & -e_{21} & -e_{31}\\ C_{21}^{E} & C_{22}^{E} & C_{23}^{E} & 0 & 0 & C_{26}^{E} & -e_{12} & -e_{22} & -e_{32}\\ C_{31}^{E} & C_{32}^{E} & C_{33}^{E} & 0 & 0 & C_{36}^{E} & -e_{13} & -e_{23} & -e_{33}\\ 0 & 0 & 0 & C_{44}^{E} & C_{45}^{E} & 0 & -e_{14} & -e_{24} & -e_{34}\\ 0 & 0 & 0 & C_{54}^{E} & C_{55}^{E} & 0 & -e_{15} & -e_{25} & -e_{35}\\ C_{61}^{E} & C_{62}^{E} & C_{63}^{E} & 0 & 0 & C_{66}^{E} & -e_{16} & -e_{26} & -e_{36}\\ e_{11} & e_{12} & e_{13} & e_{14} & e_{15} & e_{16} & \chi_{11}^{S} & \chi_{12}^{S} & 0\\ e_{21} & e_{22} & e_{23} & e_{24} & e_{25} & e_{26} & \chi_{21}^{S} & \chi_{22}^{S} & 0\\ e_{31} & e_{32} & e_{33} & e_{34} & e_{35} & e_{36} & 0 & 0 & \chi_{33}^{S} \end{array}\right]\left\{\begin{array}{c} \varepsilon_{xx}\\ \varepsilon_{yy}\\ \varepsilon_{zz}\\ \varepsilon_{xz}\\ \varepsilon_{yz}\\ \varepsilon_{xy}\\ E_{x}\\ E_{y}\\ E_{z} \end{array}\right\}$$

For more information about the two-dimensional electromechanical model, refer to [34].

### 2.2. Plate Elements with Higher Order Kinematics

**Figure 2:**
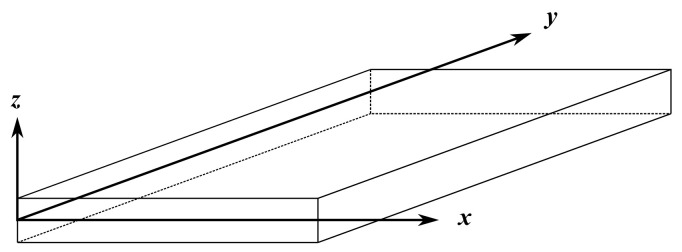
Geometry and reference system of a 2D plate model.

Refined two-dimensional plate electro-mechanical models are presented in this section. The reference coordinate system is as shown in Figure 2. Plate models in the mechanical case can be refined using the CUF by expanding a generic function $F_{\tau }\left(z\right)$ defined to the thickness of the plate, resulting in the following expression:

(9) $$q=F_{\tau }\left(z\right)q_{\tau }\left(x,y\right)\;\;\tau =0,\cdots ,N.$$

where *N* represents the number of expansion terms. $q={\left\{u,v,w,\phi \right\}}^{T}$ is the generalized displacement vector, and $q_{\tau }\left(x,y\right)$ is the planar unknown vector defined on the neutral plane of the plate.

Various theories can be used when defining the through-the-thickness functions $F_{\tau }\left(z\right)$. In the analysis of multi-layered structures, Equivalent Single Layer (ESL) approaches use Taylor-like expansions, while Layer-Wise (LW) models adopt Lagrange-type expansions. When CUF-based functions are applied to the thickness to formulate the plate elements, the inplane solution $q_{\tau }\left(x,y\right)$ can be approximated by the Lagrangian shape functions $N_{i}\left(x,y\right)$ as follows:

(10) $$\begin{array}{c} q=N_{i}\left(x,y\right)F_{\tau }\left(z\right)q_{i\tau }\left(x,y\right)\\ \;\;\tau =0,\cdots ,N;i=1,\cdots ,M. \end{array}$$

where $F_{\tau }$ represent the node kinematics of a plate element.

#### 2.2.1. Taylor Expansions (TE)

Taylor series are used to build the through-the-thickness functions $F_{\tau }\left(z\right)$ of TE-type kinematics, where the series are taken as $z^{m}$ (where *m* is a positive integer). For example, the displacement field based on the third-order TE expansions can be expressed as:

(11) $$\left\{\begin{array}{cc} u & =F_{1}u_{1}+F_{2}u_{2}+F_{3}u_{3}+F_{4}u_{4}\\ v & =F_{1}v_{1}+F_{2}v_{2}+F_{3}v_{3}+F_{4}v_{4}\\ w & =F_{1}w_{1}+F_{2}w_{2}+F_{3}w_{3}+F_{4}w_{4}\\ \phi & =F_{1}\phi_{1}+F_{2}\phi_{2}+F_{3}\phi_{3}+F_{4}\phi_{4} \end{array}\right.$$

where $F_{\tau }$ holds the following terms:

(12) $$\begin{array}{cc} \; & F_{1}=1,\;F_{2}=z,\\ \; & F_{3}=z^{2},\;F_{4}=z^{3} \end{array}$$

In this Framework, “TE*n*” refers to kinematics based on Taylor series, where *n* indicates the highest order of the adopted polynomial. Classical Plate Theory (CPT) and First-order Shear Deformation Theory (FSDT) can be treated as a particular case of a TE model, with the terms *n* = −1 and 0, respectively.

#### 2.2.2. Lagrange Expansions (LE)

LE-type kinematics can be constructed by using Lagrange interpolation polynomials as the through-the-thickness functions. Taking the Lagrange interpolation polynomials on the top and bottom points of a linear element (B2) as an example, the expansion can be expressed as follows:

(13) $$\left\{\begin{array}{cc} u & =F_{1}u_{1}+F_{2}u_{2}\\ v & =F_{1}v_{1}+F_{2}v_{2}\\ w & =F_{1}w_{1}+F_{2}w_{2}\\ \phi & =F_{1}\phi_{1}+F_{2}\phi_{2} \end{array}\right.$$

where $\left(u_{1},v_{1},w_{1},\phi_{1}\right)$ and $\left(u_{2},v_{2},w_{2},\phi_{2}\right)$ are the displacement components at the top and the bottom of the plate, respectively, and $F_{1}$, $F_{2}$ are linear Lagrange functions expressed as follows:

(14) $$\begin{array}{cc} \; & F_{1}=\frac{1+\zeta }{2}\\ \; & F_{2}=\frac{1-\zeta }{2} \end{array}$$

where −1<ζ<1, and where the functions *F* are used in the natural thickness coordinate of the plate. Note that the Lagrange functions have the following unique properties:

(15) $$\begin{array}{ccc} \; & F_{1}=1, & F_{2}=0\;for\;\zeta =1\\ \; & F_{1}=0, & F_{2}=1\;for\;\zeta =-1 \end{array}$$

When using displacement-based LE models, the degrees of freedom of the FEM models are the actual physical translational displacements of the through-the-thickness nodes. Using LE expansions provides continuity of transverse shear stresses at the interfaces and permits the approximation of zig-zag distribution of shear deformation. For more details, refer to [35]. In the work of this paper, the mentioned through-the-thickness expansion B2, B3 and B4 elements represent the linear, quadratic and cubic expansion elements, respectively.

### 2.3. Node-Dependent Kinematic (NDK) for Pate Elements

As mentioned before, when using CUF plate formulation, the displacement vector q(x,y,z) can be split into two contributions, the through-the-thickness approximation $F_{\tau }\left(z\right)$ controlling the kinematics, and the in-plane solution $q_{\tau }\left(x,y\right)$ which can be approximated using the Lagrangian shape functions $N_{i}\left(x,y\right)$. When each node has a different kinematic, the displacement field can be written as follows:

(16) $$q=N_{i}\left(x,y\right)F_{\tau }^{i}\left(z\right)q_{i\tau }\left(x,y\right)\;\tau =0,\cdots ,N;i=1,\cdots ,M.$$

**Figure 3:**
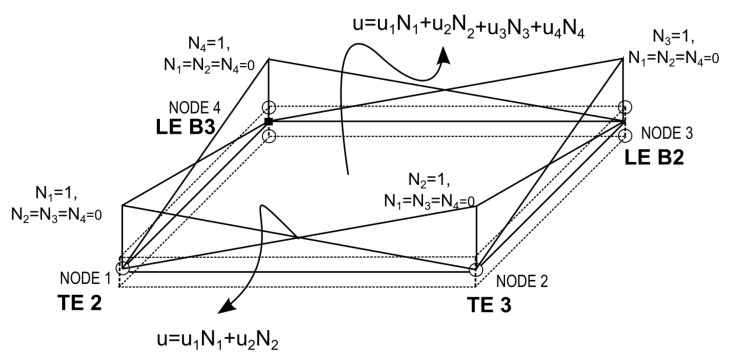
Displacement in Q4 plate element with node- dependent kinematics.

Using the FEM Lagrangian shape functions, it is possible to interpolate individually defined nodal kinematics over the neutral plane of a plate element, obtaining elements with variable LW/ESL capabilities from node to node, depending on the kinematics used at each node. Figure 3 presents an example of a plate element of four nodes, Q4, formulated with a node-dependent kinematic, where at node 1 and node 2 different TE kinematics are used, while at node 3 and node 4 different LE-type functions (linear, B2, and quadratic, B3) for through-the-thickness are used. For LE Kinematics, the DOF at the nodes depends on the order of expansion to the thickness. At node 3, the number of unknowns is 6 which corresponds to the three variables at each node of the B2 element (top and bottom), whereas at node 4 the number of unknowns is nine. As the number of layers increases in the model, the number of unknowns increases. In contrast, the number of unknowns in the TE models only depends on the order of the used Taylor functions. For example, in a mechanical problem, using TE2 and TE3 in node 1 and node 2 would result in nine and twelve unknowns, respectively, at each corresponding node. The displacement inside the element is a linear combination between the different nodal displacements.

These elements provide the connection between nodes with different kinematics, connecting a higher order and a lower order model, thus enabling the local refinement of the model, and thus decreasing the computational cost of a fully refined model.

### 2.4. Dynamic Model

For a dynamic problem, the general displacement vector of Equation (16) can be written as follows:

(17) $$q=F_{\tau }\left(z\right)q_{\tau }\left(x,y,t\right)\;\;\tau =0,\cdots ,N.$$

The displacement $q_{\tau }$ is then interpolated along the plate neutral plane, using the shape functions in the x-y plane, as follows:

(18) $$q_{\tau }\left(x,y,t\right)=N_{i}\left(x,y\right)q_{i\tau }\left(t\right)$$

The general stress vector $\bar{\sigma }$ and strain $\bar{\varepsilon }$ can the be written as follows:

(19) $$\bar{\sigma }=\tilde{H}DF_{\tau }N_{i}q_{i\tau }\;\;\bar{\varepsilon }=DF_{\tau }N_{i}q_{i\tau }$$

By applying the principle of virtual displacement (PVD), the governing equations can be derived. Substituting the constitutive equations, we obtain the following expression:

(20) $$\delta L_{ine}+\delta L_{int}=\delta L_{ext}$$

where $L_{ine},L_{int}$ and $L_{ext}$ represent the work performed by inertia loads, internal work, and the work performed by external forces, respectively, where δ stands for virtual variation. Neglecting damping, the above equation can be further written as:

(21) $$\int_{V}\rho \delta q^{T}\ddot{q}dV+\int_{V}\delta {\bar{\varepsilon }}^{T}\bar{\sigma }dV=\int_{V}\delta q^{T}\bar{P}dV$$

If the geometrical relations and shape functions in Equation (19) are substituted into the above expression, we can obtain the following in an abbreviated form:

(22) $$\delta q_{js}^{T}m_{ij\tau s}{\ddot{q}}_{i\tau }+\delta q_{js}^{T}{\tilde{k}}_{ij\tau s}q_{i\tau }=\delta q_{sj}^{T}{\bar{P}}_{sj}$$

where $m_{ij\tau s}$ and ${\tilde{k}}_{ij\tau s}$ are the 4 × 4 fundamental nuclei of the mass stiffness matrices, respectively, and ${\bar{P}}_{sj}$ is a 4 × 1 load vector. In an extended format, they can be defined as follows:

(23) $$\begin{array}{cc} \; & m_{ij\tau s}=\int_{V}\rho N_{i}F_{s}^{j}N_{j}F_{\tau }^{i}dV\\ \; & {\tilde{k}}_{ij\tau s}=\int_{V}N_{j}F_{s}^{j}D^{T}\tilde{H}DF_{\tau }^{i}N_{i}dV\\ \; & {\bar{P}}_{sj}=\int_{V}F_{s}^{j}N_{j}\bar{P}dV \end{array}$$

Thus, the following governing equations can be obtained for a system without damping:

(24) $$M\ddot{Q}+KQ=F$$

where M and K are the global mass and stiffness matrices, respectively, assembled from the fundamental nuclei and Q is the unknowns vector.

For additional information regarding the components of the individual terms in the fundamental nucleus of the stiffness matrix, refer to [34].

For Dynamic problems, the above Equation (24) needs to be discretized in time. The used scheme is the same implicit time scheme used by [32], as the goal was to compare the different theories, and the advantages of using an explicit dynamic model were lost as diagonalizing the mass matrix is not always achievable. As implicit models are computationally expensive, it is possible to reduce the computational time by deriving the mass and stiffness matrices only once, as they do not change in a linear problem. The displacement and acceleration equations adopted in the model were those proposed by the implicit Newmark method. The velocities and accelerations at time t+Δt are defined by:

(25) $$\begin{array}{cc} \; & {\dot{Q}}_{t+\Delta t}={\dot{Q}}_{t}+\left[\left(1-\gamma \right){\ddot{Q}}_{t}+\gamma {\ddot{Q}}_{t+\Delta t}\right]\\ \; & Q_{t+\Delta t}=Q_{t}+{\dot{U}}_{t}\Delta t+\left[\left(1/2-\beta \right){\ddot{Q}}_{t}+\beta {\ddot{Q}}_{t+\Delta t}\right]\Delta t^{2} \end{array}$$

where γ and β are parameters used to control the stability of the model.

## 3. Mechanical Benchmark Problem

**Figure 4:**
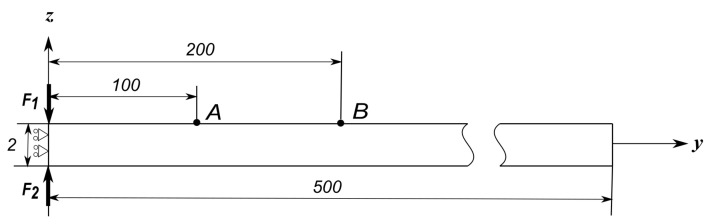
Boundary conditions and geometry of the mechanical plate model adopted for the propagation of Lamb waves, dimensions in mm.

In order to model the Lamb wave propagation, a simple benchmark problem was adopted from [33]. The described model consists of an isotropic aluminium strip. The mechanical properties of the material used can be found in Table 1. The length of the strip is l=500 mm, with a thickness h=2 mm. The assumed width was chosen to be w=10 mm to facilitate the unidirectional propagation of the applied signal, while limiting the number of plate elements to the width to a unit mesh element. Figure 4 shows the geometry and the applied boundary conditions. At y=0, the plane of symmetry in the y direction was imposed. Similarly, two planes of symmetry in the x direction were imposed at the boundaries for x=0 and x=10 mm. The mechanical loads were applied at the top and bottom nodes at the position y=0, as shown. The analysis was carried out assuming no material damping.

The applied forces $F_{1}$ and $F_{2}$ are time dependent. They follow a modulated sinusoidal burst signal presented in Equation (26), where ω=2πf represents the central frequency, and *n* denotes the number of cycles within a signal.

(26) $$F\left(t\right)=\tilde{F}sin\left(\omega t\right)sin^{2}\left(\frac{\omega t}{2n}\right)$$

**Figure 5:**
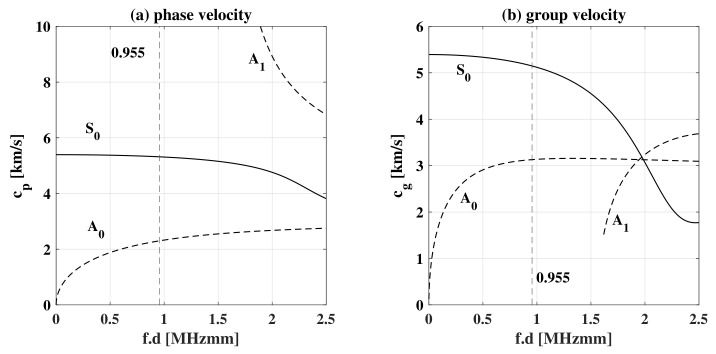
Dispersion curves of 2 mm thickness aluminium plate plotted using DC v2.0. The phase velocity curves are plotted to the left, whereas the group velocity curves are plotted on the right.

**Figure 6:**
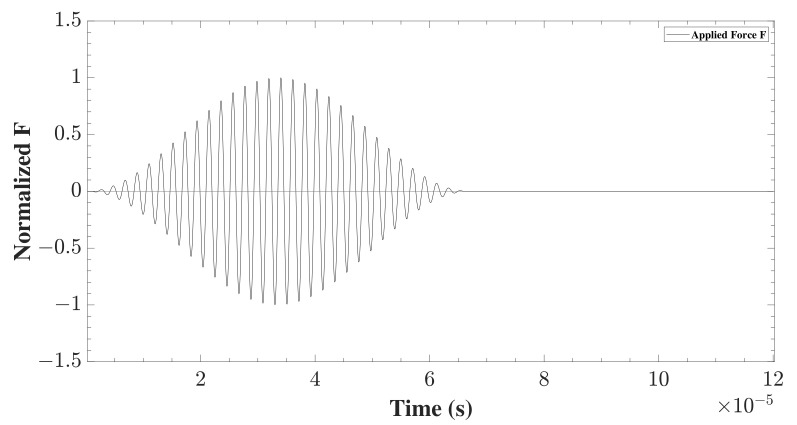
The shape of the applied modulated force, plotted and normalized as a function of time.

The value of *n* determines the width of the excited frequency band around the central frequency. In this study, n=32 was chosen to be rather high, to ensure the frequency width was narrow-banded around the central frequency. The choice of the central frequency is influenced by the dispersion curves of the material, see Figure 5. The aim was to excite the mono-modal symmetric ($S_{0}$) and antisymmetric ($A_{0}$) fundamental modes, without exciting the other higher order modes. This is achievable when the frequency-thickness coefficient fd<1.5 MHzmm. The central frequency f=477.5 kHz was chosen as the dispersion effect is relatively low for both models at this frequency. The shape of the force modulation can be seen in Figure 6. Both forces applied were of the same magnitude in time. The direction of the applied forces determines which kind of wave is generated. If the forces are opposite to one another, $S_{0}$ waves are generated. In contrast, if they have the same direction, the $A_{0}$ mode is generated.

### 3.1. Evaluation Criteria

**Figure 7:**
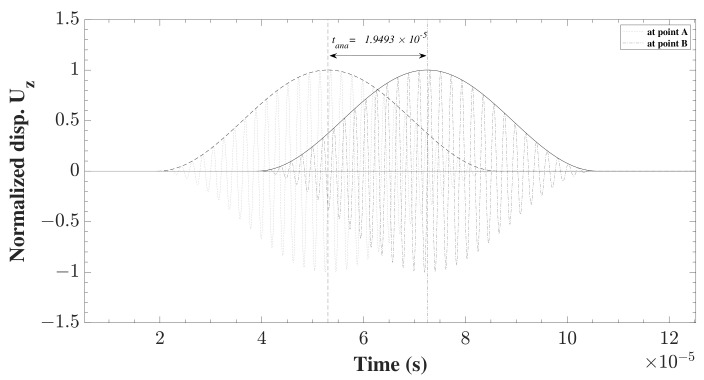
Analytical time-of-flight $t_{ana}$ of $S_{0}$ wave propagating between A and B.

In order to adopt a uniform technique for the evaluation of the quality of the different models under different assumptions and changing parameters, a methodology needed to be proposed. The used methodology is the one mentioned in the above reference. The propagation of the wave was monitored between the two points of A and B, and the time-of-flight between the two points was used. The numerical value, $t_{num}$, was compared to the analytical time, $t_{ana}$. In this study, the analytical time was computed form the group velocity of the propagating waves given by [32] extracted from the analytical solution of the dispersion curves, see Equation (27). Then, the propagating wave was shifted by a time corresponding to the above calculated analytical group velocity multiplied by the distance covered. Figure 7 shows the $t_{ana}$ for the propagating $S_{0}$ waves between points A and B.

(27) $$\frac{tan(\beta \frac{d}{2})}{tan(\alpha \frac{d}{2})}={\left[\frac{4\alpha \beta k^{2}}{{\left(k^{2}-\beta^{2}\right)}^{2}}\right]}^{a}$$

where a=+1 or −1 to obtain the symmetric or antisymmetric mode, respectively, $\alpha^{2}=\frac{\omega^{2}}{c_{1}^{2}}-k^{2}$, $\beta^{2}=\frac{\omega^{2}}{c_{2}^{2}}-k^{2}$, with ω being the angular frequency and *k* being the wave number. These equations can be solved to provide

(28) $$c_{p}=\frac{\omega }{k}\;\;and\;\;c_{g}=c_{p}^{2}\left(c_{p}-\omega \frac{\partial c_{p}}{\partial \omega }\right)$$

As the group velocities of $S_{0}$ and $A_{0}$ modes are different, see Table 2, $t_{ana}$ is different in each of the studied cases. In order to extract this time, the envelope of the wave has to be plotted. Thus, Hilbert transformation, Equation (29), was carried out on the time signal of the displacement $u_{z}\left(t\right)$ at points A and B, respectively,

(29) $$H_{A,B}\left(u_{z}\left(t\right)\right)=\frac{1}{\pi }\int_{-\infty }^{+\infty }u_{A,B}\left(\tau \right).\frac{1}{t-\tau }d\tau$$

Using the Hilbert transform function, with the time-dependent displacement signal $u_{z}\left(t\right)$, the envelope of the signal $e_{A,B}$ was constructed at each of the points, according to Equation (30):

(30) $$e_{A,B}\left(t\right)=\sqrt{H_{A,B}{\left(u_{z}\left(t\right)\right)}^{2}+u_{A,B}{\left(t\right)}^{2}}$$

For this purpose, MATLAB R2021a was used as it provides the function which implements the previous equations and calculates the envelope of the input signal. Afterwards, the centroid of the envelope was calculated at the two points. The simple subtraction of the two centroids $t=t_{B}-t_{A}$ provides the time needed for the wave to propagate between A and B. The centroid was calculated using Equation (31):

(31) $$t_{A,B}=\frac{\int_{0}^{t_{f}}e_{A,B}\;.\;t\;dt}{\int_{0}^{t_{f}}e_{A,B}\;dt}$$

### 3.2. Model Parameters

As the aluminium strip modelled is slender, the number of mesh elements to the width was limited and fixed to a single mesh element. It was mentioned in [36] that it is sufficient to use 3 to 5 nodal points at a half wavelength for dynamic harmonic problems along the propagation direction. In [26], it was stated that element size $L_{max}$ must be less than one-tenth the wavelength (λ) for $A_{0}$ mode, with a time step (Δt), such that

(32) $$\Delta t<1/\left(20f_{max}\right)\;\;,\;\;L_{min}⩽\lambda /10$$

On the other hand, [32] specified the time step using the Courant–Friedrich–Levy condition, which states

(33) $$\Delta t⩽l_{e}/c_{g}$$

where $l_{e}$ denotes the finite element mesh size and $c_{g}$ denotes the group velocity of the propagating wave. Similar time step and element size values were mentioned in [27] while using an explicit dynamic model to ensure convergence criteria. In this study, mesh density was altered along the direction of wave propagation (y-direction) in order to determine the number of elements where the model reaches the numerical convergence. The meshes were refined gradually, starting from a course mesh of 100 elements along the length, and reaching an extremely refined mesh of 2000 elements. In some models where the computation cost is low and the convergence is slow, this number reaches 4000 elements. Aside from the in-plane mesh density, the type of plate element used was also altered. Linear 4 node plate elements (Q4), quadratic 9 node plate elements (Q9), and cubic (Q16) plate elements were investigated. The time step of the implicit dynamic problem also varied. For $S_{0}$ and $A_{0}$ wave propagation, the model time was 120 and 150 μs, respectively. This time was divided by a total number of time steps N. In this study, four values of N were used, namely 500, 1000, 2000, and 4000. Afterwards, the model kinematics (through-the-thickness expansion) were studied under a fixed plate element type and under a fixed number of time steps. As the wavelength of $S_{0}$ and $A_{0}$ modes are different, due to their different phase velocities, another parameter χ is adopted, which is defined as the number of degrees-of-freedom (DOF) in each wavelength, expressed as

(34) $$\chi_{S0,A0}=\frac{N_{DOF}}{l}\lambda_{S0,A0}=\frac{N_{DOF}\;\cdot \;c_{p_{S0,A0}}}{l\;\cdot \;f}$$

where $N_{DOF}$, λ, $c_{p}$ and *l* denote the total number of DOF, the wavelength, the phase velocity and the length of the strip, respectively. The error was calculated as the relative difference between the analytical and numerical time to flight *t* between points A and B.

(35) $$Error\;\left[\%\right]=\frac{t_{ana}-t_{num}}{t_{ana}}\times 100$$

### 3.3. Numerical Results and Discussion

**Figure 8:**
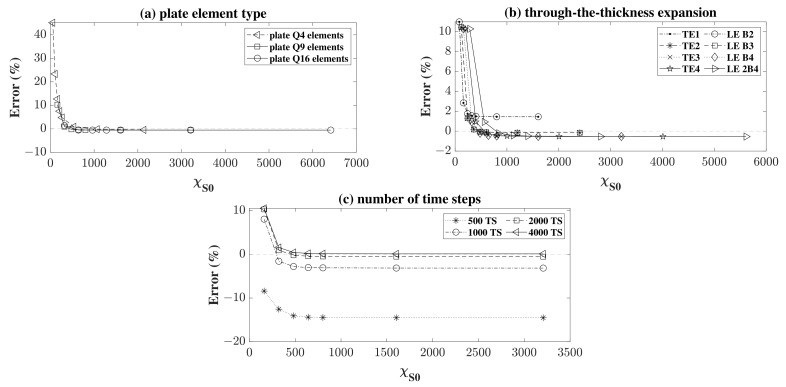
Error in (%) for (**a**) different plate element types, (**b**) different expansions through-the-thickness, and (**c**) different time step number as a function of $\chi_{S0}$.

The results of symmetric ($S_{0}$) wave propagation were first plotted in a linear scale in Figure 8 in order to provide an idea about the progression of the values of the error and the corresponding convergences obtained. Figure 8a shows the error obtained for a fixed time step number (2000 TS) and for a fixed through-the-thickness Lagrange LE B4 expansion. The convergence behaviour for the three element types can be noticed as the value of $\chi_{S0}$ increases, without any major differentiation between the tree types aside from that the error values at lower $\chi_{S0}$ are much higher when Q4 plate elements are used. Figure 8b shows the error for different t-the-thickness expansions under a fixed time step number (2000 TS) and for a fixed plate element type (Q9). It is shown that different expansions may produce a different numerical conversion. Figure 8c shows the effect of the choice of the time step (number of time steps) on the convergence of the error, under a fixed plate element type and expansion, (Q9) and (LE B4), respectively. It is shown that the more time steps, the less the error in convergence, however, on the expense of a longer dynamic numerical model analysis. In the present problem the results suggest that a value of 2000 time steps (2000 TS) or more is considered appropriate for obtaining good convergence results.

**Figure 9:**
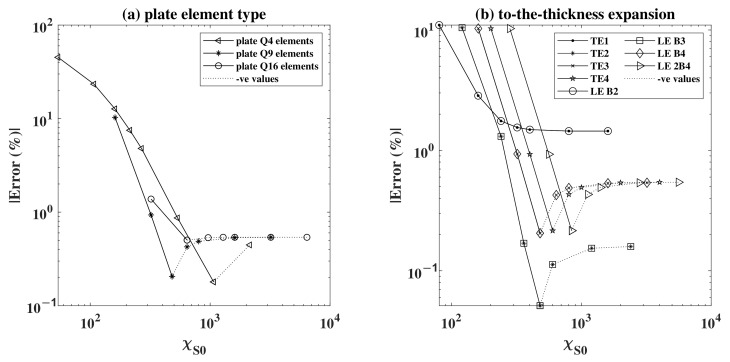
Absolute Error in (%) for (**a**) different plate element types and (**b**) different expansions through-the-thickness, in a log-log scale, as a function of $\chi_{S0}$.

For a better representation of the results in Figure 8a,b, the graphs were re-plotted by adopting a log-log scale. As some of the models converged to a negative value, the absolute value of the error, denoted by absolute error, was calculated in the graphs as shown in Figure 9 to permit the use of the log scale. This is the reason behind the sudden increase in the values after their descent, as in a linear scale the error crossed below the x-axis. The results are plotted in Figure 9a,b, respectively, showing the convergence behaviour under the effect of the plate element used and the through-the-thickness expansion, where the negative values are plotted in a different line plot. The assessment was always carried out while successively refining the mesh size, expressed in χ. In Figure 9a, it can be noticed that plate elements with higher nodes converge faster, for a lower value of $\chi_{S0}$. This shows that to achieve the accuracy produced by higher order plate elements, a much-refined mesh of a lower plate element type in needed. Using the same mesh refinement for different elements produces structures with different stiffness, where the lower the plate order is, the stiffer the structure becomes. This is shown by the higher error in the low order plate elements (Q4). Keeping in mind that χ depends on the DOF of the system, for the same mesh number we obtained a higher DOF for higher order elements, and thus higher χ.

On the other hand, in Figure 9b, it can be seen that the higher the order of expansion through-the-thickness, the more the solution converges to the analytical solution. Keeping in mind that the results were plotted for a fixed time step of 2000 TS, it is understandable why some lower order graphs seemed to converge to a lower error. This is in fact explained due to the cumulative error between the number of time steps used in the numerical model, which is negative, and that of the expansion, which is positive, as given that they are in the proper order, they cancel each other out. The reassuring result is that the higher order expansions (LE 2B4, TE4, …) converge to the same value under the imposed conditions. The higher the order, the more costly the problem is, hence a higher DOF and a higher value of $\chi_{S0}$. It can also be shown that the TE1 and LE B2 expansions give the same results. The same can be said for TE2 and LE B2 and for TE3 and LE B4. This is explained by the fact that for a single layered structure, the first order Taylor and linear Lagrange polynomials provide the same formulation with the same number of DOF. The only difference is that it is much easier to impose the displacement boundary conditions for Lagrange elements as the unknowns of the problem are directly the displacements at the nodes. The same explanation can be adopted for the concatenating results of second and third order Taylor with the quadratic and cubic Lagrange, respectively.

**Figure 10:**
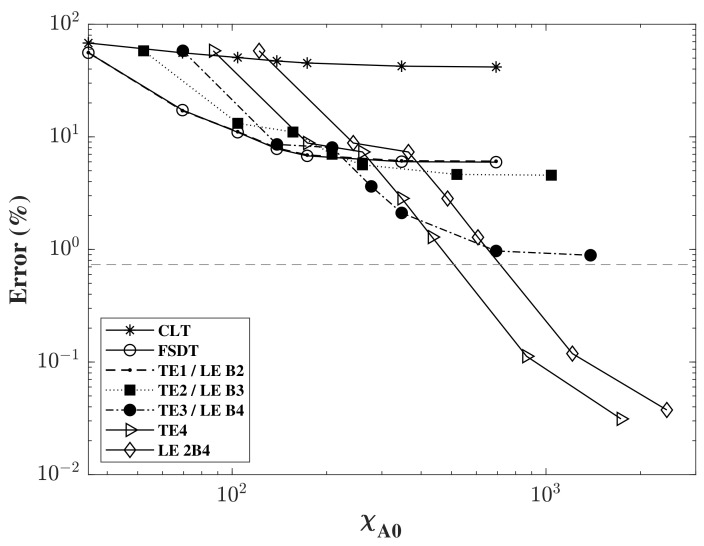
Error in (%) of $A_{0}$ wave propagation for different expansions through-the-thickness, in a log-log scale, as a function of $\chi_{A0}$.

Consequently, the same analysis was performed for the propagating $A_{0}$ Lamb wave, however, the analysis was deemed similar to the previous one in terms of the plate element type and the number of time steps used. Thus, the model was limited to the study of convergence under different model kinematics, with different in-plane mesh refinements under an increased fixed predetermined time-step of 4000 steps in a model time of 150 μs and with a nine-node plate element mesh type (Q9). The results were plotted in Figure 10, as the error was computed and plotted as a function of $\chi_{A0}$. As the results of [31,32,33] suggest, it was expected that the higher order expansion to the thickness would produce a lower error, as the convergence values decrease for an increased order of expansion. It can also be seen that the convergence in the $A_{0}$ case was slower than that of the $S_{0}$ case, where increased mesh refinement was needed to reach convergence. In the graph, the results were plotted as a function of $\chi_{A0}$, which is lower than that of $\chi_{S0}$ for the same mesh number due to the lower phase velocity of the antisymmetric wave. It is also noticeable that there were no negative error values in this study, as the number of time steps was high, producing a very refined time step (Δt), thereby minimizing the error resulting from the choice of Δt.

### 3.4. Impact of the Kinematic Model

**Figure 11:**
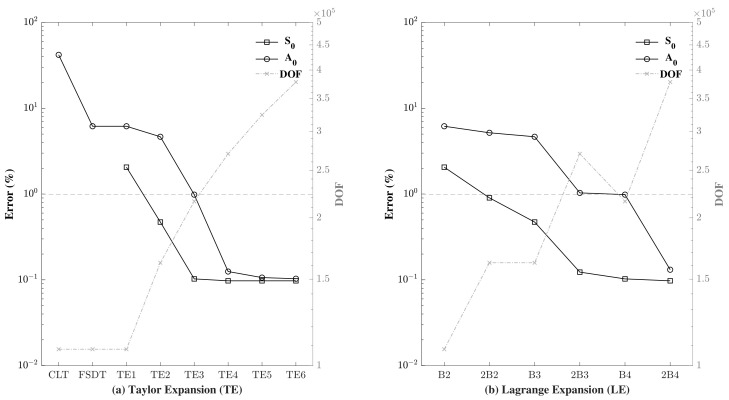
Assessment of the symmetric $S_{0}$ and the antisymmetric $A_{0}$ Lamb wave propagation under different through-the-thickness orders of expansion, in both (**a**) Taylor and (**b**) Lagrange models.

Accordingly, and in order to compare the implementation of the different orders of transverse expansion, for both $S_{0}$ and $A_{0}$, a refined mesh model was implemented with a fixed in-plane mesh of 3000 nine-node (Q9) plate elements for the length. This was to ensure numerical convergence of the obtained results. Furthermore, in order to properly compare the numerical model with the analytical model, a refined time step was adopted. To achieve this, 4000 time steps were implemented for the $S_{0}$ problem in a model time of 120 μs, and 5000 steps were implemented for the $A_{0}$ problem for a model time of 150 μs, obtaining the same time step Δt for both problems. Under these parameters, the error was calculated and the different through-the-thickness expansion models were compared. Figure 11 shows the results of the assessment. Admitting a 1% error or less as an acceptable result, it can be noticed that the $A_{0}$ propagation certainly requires higher order through-the-thickness expansions in order to reach the allowed limit. For Taylor expansion, a third order expansion TE3 or more is needed for the $A_{0}$ problem, whereas Taylor to the second order is sufficient for the $S_{0}$ problem. Note that the classical theories were obtained from the first order Taylor expansion TE1 by applying some constraints. This is the reason behind the observation of the same DOF for these theories with the TE1. Unlike the $A_{0}$ problem, where the wave propagation occurs due to the shear strain, modelling the $S_{0}$ problem using the classical theories does not generate any results as displacements to the thickness are constant in these models. The same can be said about Lagrange expansion models, where higher order models are needed with one or more B4 elements to the thickness to model $A_{0}$ wave propagation; conversely, a much lower number of expansions are sufficient for $S_{0}$ wave propagation. It can also be observed that the use of one higher order element through-the-thickness (B4, B3) generates better results than the use of two lower order elements (2B3, 2B2), even though the DOF of the latter is greater than (or sometimes equal to) that of the former. For example, 2B2 splits the thickness in half and produces linear displacements in each half, whereas B3 describes the quadratic behaviour of the displacement through the thickness, which cannot be described by two linear elements. These results are in contrast to those obtained by [31,32], with the major difference that the authors in the literature applied the higher order models for wave propagation in a 1D beam model, whereas a plate model was used in our study.

### 3.5. Node-Dependent Kinematics Models

The implementation of NDK is introduced in this section in order to study the reduction in the computational cost of the dynamic wave propagation model and the effect on the accuracy of the model. The case adopted was the simple mechanical benchmark described in previous sections. The strip was divided into 2000 Q9 mesh elements in length, and the dynamic analysis was performed in a fixed 4000 time steps in a model time of 150 μs for both $S_{0}$ and $A_{0}$ problems.

**Figure 12:**
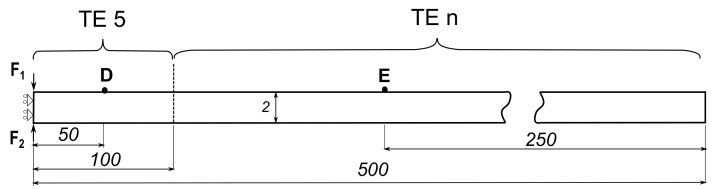
The adopted NDK model showing the model kinematics and the boundary conditions, with the dimensions provided in mm.

As shown in Figure 12, fixed TE5 kinematics were adopted in the first 100 mm of the strip, whereas for the rest of the strip a node-dependent kinematic model was adopted based on Taylor expansion of different orders, denoted as TEn, where n describes the order of the model used. On the other hand, the reference that was adopted in the study was a model with a full Taylor expansion to the fifth order (TE5). The error was computed in the same manner as before, expressed in Equation (36), by the time needed for the wave to travel from point D to point E for the NDK model compared to that of a full TE5 expansion model.

(36) $$Error\;\left[\%\right]=\frac{t_{TE5}-t_{NDK}}{t_{TE5}}\;\cdot \;100$$

**Figure 13:**
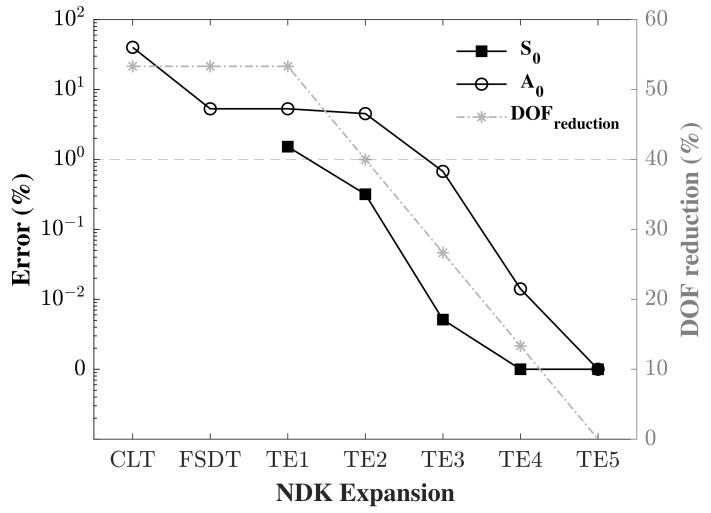
Assessment of the error in (%) in a node-dependent kinematics model, with $S_{0}$ and $A_{0}$ wave propagation, compared to a full TE5 model.

The error was calculated and was plotted alongside the DOF of the respective model in the same graph and in a log scale. Figure 13 shows the error for both $S_{0}$ and $A_{0}$ cases and the DOF according to the TEn model adopted. CLT and FSDT denote the Classical Laminate Theory and the First order Shear Deformation Theory, respectively. The Expansion orders on the abscissa refer to the order adopted in the respective NDK model.

It is clearly shown that using a low order model coupled with a high order model reduces the accuracy, and the lower the NDK model is the higher the error obtained. Assuming a 1% error acceptable in these conditions, it can be seen that in the case of $S_{0}$, it is possible to couple second order Taylor expansion or Taylor expansion of a higher order to TE5 and produce acceptable results. When TE2 is chosen, the decrease in the DOF of the model is calculated to be around 40%. In the case of $A_{0}$ wave propagation, in order to obtain satisfactory results, the NDK model cannot be selected as lower than TE3, thus saving 27% of the computational cost. Using the CLT is strongly advised against as it generates a high error. Table 3 shows the results of the NDK analysis of the mechanical case, with the calculated time, error (%), DOF and DOF reduction (%) for both cases. It was noticed that in $S_{0}$, the use of TE4 instead of TE5 produced zero errors with a lower computational cost.

## 4. Electromechanical Coupled Case: Actuation and Sensing Using PZT

**Figure 14:**
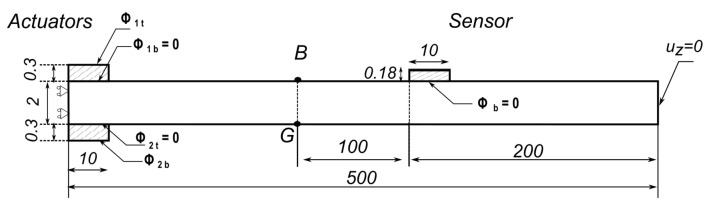
Benchmark of the coupled SHM problem, with the applied boundary conditions, and dimensions (in mm).

To achieve a more realistic case of structural health monitoring (SHM) of the isotropic aluminium strip, a piezo-electrical coupled analysis was conducted. Piezo-electric transducers (PZT-5A) were used instead of applying a force and reading the displacement from the nodes. Two piezoelectric transducers with the properties specified in Table 4 were placed on the top and bottom of the structure, as actuators, and another PZT acting as a sensor was placed on the top. Figure 14 shows the positioning and dimensions of the added transducers.

The Lamb wave was generated by applying a modulated electric potential difference Φ on the actuator transducers, using the same shape as the force used in the previous section, with the same values of the parameters as the analysis was performed on the same structure, and where:

(37) $$\Phi \left(t\right)=\tilde{\Phi }sin\left(\omega t\right)sin^{2}\left(\frac{\omega t}{2n}\right)$$

**Figure 15:**
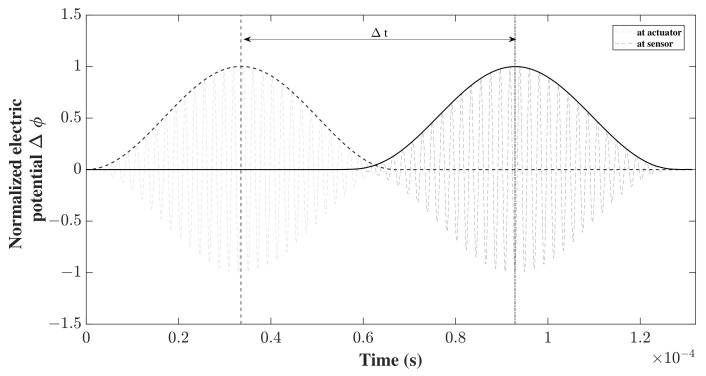
Time-of-flight Δt of $S_{0}$ wave propagating, obtained from the electric potential (ΔΦ) between sensor and actuator.

The applied potential difference on the upper and lower transducers, respectively, is between the potential of upper and lower electrodes $\Phi_{1,2}=\Phi_{1,2t}-\Phi_{1,2b}$. For the symmetric $S_{0}$ case, $\Phi_{1}=\Phi_{2}$, whereas $\Phi_{1}=-\Phi_{2}$ for the antisymmetric $A_{0}$ case. The electric potential difference at the middle top of the PZT sensor was measured, where the top of the sensor was covered by a thin layer of negligible stiffness and of high conductivity acting as an electrode. The model time was chosen to be slightly longer in this case as the wave was required to travel at least 300 mm. It was set as 150 μs, 180 μs for $S_{0}$, $A_{0}$ with 4000 and 4800 time steps, respectively, maintaining the same time step for both cases. The results were collected as the time between the applied potential difference at the actuators and the acquired potential difference at the sensor in the same manner that was performed previously for the displacements, as shown in Figure 15.

**Figure 16:**
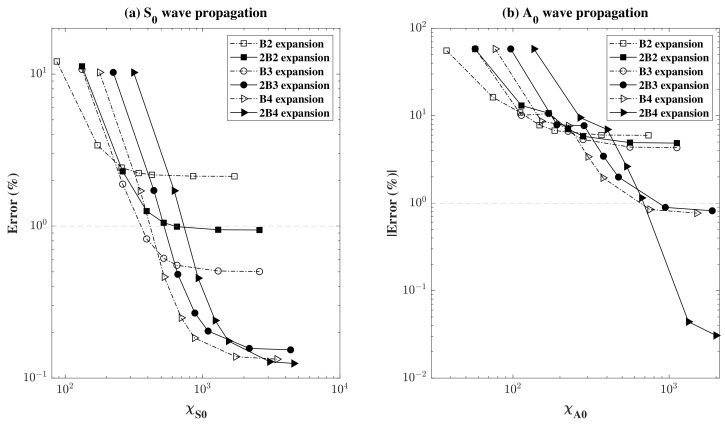
Error in (%) plotted in a log-log scale, for (**a**) $S_{0}$ and (**b**) $A_{0}$ wave propagation, using PZT actuators and sensor, as a function of χ, for different model kinematics.

These results were compared with the analytical time needed for a wave to cross a distance of 305 mm using the analytical group velocities of both waves. As the actual distance travelled might be obscure in the presence of the transducers given that they have a certain dimension along the propagation direction, an equivalent arbitrary distance was chosen between the middle of the actuator and the end of the sensor equal to 305 mm, and the numerical error was computed as before by comparing the numerical time obtained with the assumed analytical time. As described previously, the errors obtained as percentages for both fundamental cases were plotted, according to different mesh densities and different model kinematics. In this SHM problem, only the through-the-thickness kinematics based on Lagrange models were compared, as the use of Lagrange models is essential in our mixed model in order to apply the appropriate boundary conditions. The results were plotted in Figure 16 with a log–log scale. In Figure 16a,b, it can be also noticed that the model converged to lower error values with higher model kinematics. In the propagation of $A_{0}$ waves, however, some instances were of a negative value, particularly those where the mesh density was 200 elements to the length, thus the error was plotted in terms of the absolute value to favour the use of the log scale. It can be seen in Figure 16b that the convergence was not as smooth at the beginning, as the curve made a step. Checking the wave propagation at these instances (for 200 mesh elements), it can be seen that the wave travels in an unconventional way, at such a high speed that the whole structure starts to oscillate, indicating that these parameters should be avoided. Examining and comparing the results between the two wave modes, the same behaviour mentioned in the mechanical case above can be noticed. The convergence for a low error of the $S_{0}$ wave occurs for lower model kinematics, whereas higher kinematics are needed to further reduce the error in $A_{0}$ wave propagation.

**Figure 17:**
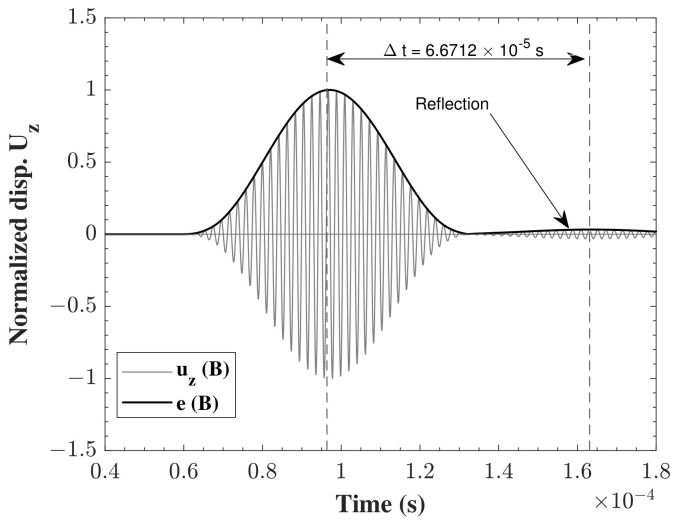
Normalized displacement $u_{z}$ at point B (5, 200, 1) under $A_{0}$ propagation.

**Figure 18:**
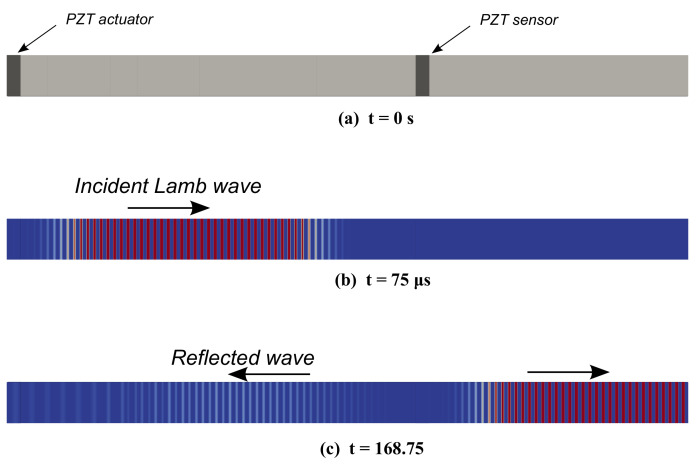
Showing (**a**) the electro-mechanical model, (**b**) incident $A_{0}$ Lamb wave and (**c**) the reflected wave.

As the wave propagates, due to the variation of geometry resulting from the positioning of the surface sensor, reflection is expected occur. Indeed, a reflection can be noticed in Figure 17 at the top surface point B of y = 200 mm. After subtracting the time at the maxima of the reflection and the propagating lamb wave at point B, and multiplying the acquired time by the corresponding group velocity, a distance of almost 200 mm (208.5 mm) can be obtained, which is equivalent to double the distance from point B to the sensor, with some differences that can be attributed to the change in the velocity of the reflected wave, or to the inaccuracy of the distance travelled of the sensor at a certain dimension. For further investigation, the figure showing the deformation of the structure displays results at different times before and after the wave encounters the sensor Figure 18b,c. It is clear that the additional wave seen in the z-displacement of point B in Figure 17 is actually the reflected part of the wave after travelling from point B, encountering the sensor, and reflecting back to point B which has a smaller amplitude.

**Figure 19:**
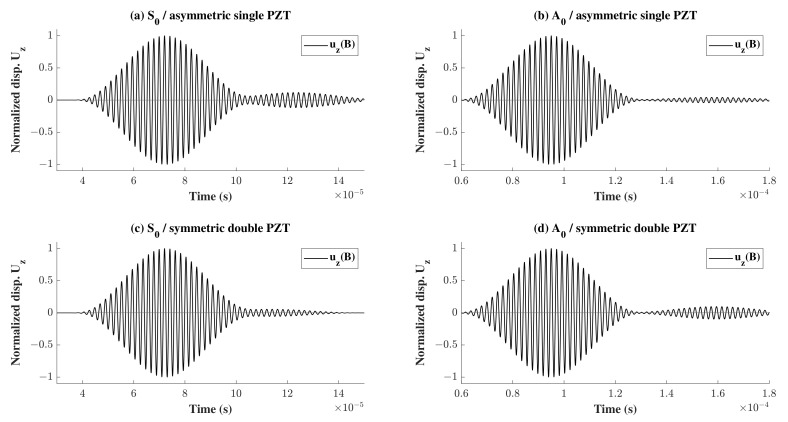
Showing the wave propagation in the the electro-mechanical model at point B, for different wave natures and different sensor configurations.

**Figure 20:**
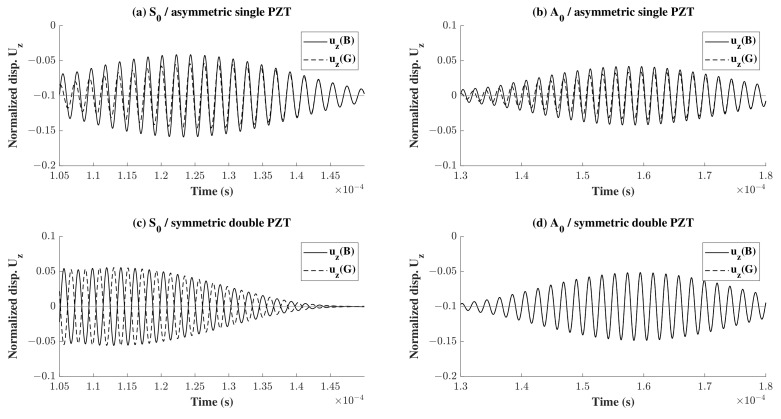
Showing a focus on the reflected waves shown in Figure 19 for the four different cases.

For further investigation, the propagating waves were plotted at point B, in Figure 19 for different wave propagation scenarios where $S_{0}$ and $A_{0}$ waves were used in a single asymmetric sensor or double symmetric surface mounted sensors. To obtain more information about the nature of the reflected wave, the z-displacement of the reflected portion of the waves at the surface points B and G was plotted for both $S_{0}$ and $A_{0}$ waves in Figure 20a,b, respectively. It can be noticed in Figure 20a that the symmetric wave gradually transforms into an antisymmetric wave, whereas in Figure 20b, although there is some phase shift at first, the wave is antisymmetric at the end. This is due to the asymmetric nature of the placement of a PZT sensor on the top. To reinforce the hypothesis, another model was considered where two sensors were placed symmetrically. The reflection was taken as described previously for both types of waves and the z-displacement was plotted at the top and bottom points B and G in as shown in Figure 20c,d. As predicted, there was no change in the nature of the reflected wave where an $S_{0}$ wave remained $S_{0}$ and vice-versa. It is also worth mentioning that comparing the plots in Figure 20 vertically for (a) and (c), it is clear that the wave is slower in case of one sensor on top compared to two sensors for $S_{0}$ wave propagation. This is not true for $A_{0}$ wave propagation as the two plots (b) and (d) look similar.

It can also be noticed that there is a change in the amplitude of the reflected wave. Comparing sub-plots (c) and (d), it can be seen that in the case of $S_{0}$, the reflected wave has a 5% amplitude of the highest displacement whereas in the case of $A_{0}$ that amplitude is of 10%. However, the opposite can be seen in sub-plots (a) and (b).

### Implementing Node-Dependent Kinematics NDK

In this section, NDK was introduced, where the areas under the PZT were modelled using a Lagrange model, and the rest of the structure was modelled with different Taylor expansion models. This was performed in order to study the effect of NDK on the error induced when attempting to reduce the computational cost of the coupled electro-mechanical model. The adopted case was the electro-mechanical benchmark described in the section above. The strip was divided into 2000 Q9 mesh elements in length, and the dynamic analysis was performed with 4000 and 4800 time steps corresponding to a model time of 150 μs for $S_{0}$ and $A_{0}$ wave propagation.

**Figure 21:**
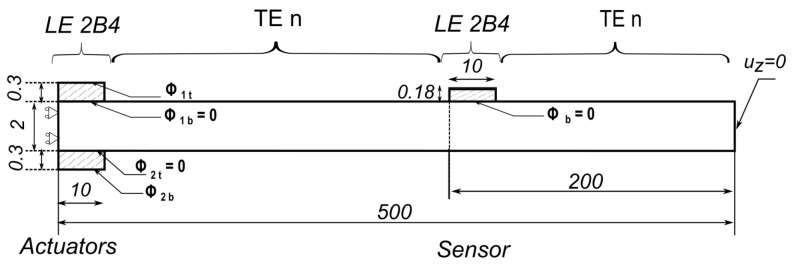
Electro-mechanical NDK model adopted, showing the model kinematics and the boundary conditions, with the dimensions given in mm.

Figure 21 shows the different kinematics used in each part of the NDK model. The actuator part consists of three layers (two PZT actuators and the strip). The model kinematics at each of those layers was fixed at LE 2B4, meaning two B4 elements were used for the thickness in each element. The same was performed in the sensor area, where it was composed of three layers, the strip, the PZT sensor and a very thin conductive material at the top of the sensor of negligible stiffness acting as an electrode. The model kinematics of the strip between the sensor and actuator and at the end of the sensor were modelled based on Taylor expansion of different orders, denoted as TEn, where n described the order of the model used. A full Lagrange expansion (LE 2B4) was adopted as a reference for both symmetric and antisymmetric waves, with which the results were compared, the error was calculated and whose DOF was reduced.

**Figure 22:**
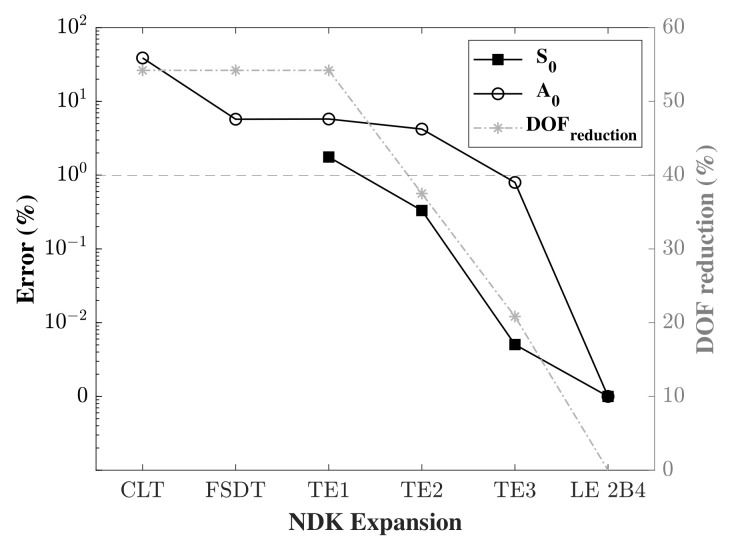
Assessment of the error in (%) in a node dependent kinematics model, with $S_{0}$ and $A_{0}$ wave propagation in an electro-mechanical plate model, compared to a refined full LE 2B4 model.

The results of the errors obtained together with the computational cost and DOF reduction are reported in Table 5 and plotted in Figure 22 according to the corresponding kinematics used in the plate region away from the actuators and sensor. It can be clearly shown that increasing the model kinematics decreased the obtained error and also decreased the level of computational cost reduction. It is also evident that using TE2 model kinematics in an $S_{0}$ wave is sufficient to produce an error of less than 1% (0.332%) with a computational cost reduction of almost 40% (37.5%). As before, the use of a more refined model is needed to achieve an error of less than 1% in $A_{0}$ wave propagation. This was attained with the use of TE3 model kinematics, which in turn led to a 20% computational cost reduction.

## 5. Conclusions

In this paper, fundamental $S_{0}$ and $A_{0}$ Lamb wave propagation were studied, propagating in a slender isotropic aluminium strip using a plate model in both the pure mechanical and coupled electromechanical cases. The model used was the MUL2 plate model, based on the Carrera Unified Formulation CUF, which was developed by the MUL2 group. Higher order models, plate element types and the number of time steps of the dynamic model were all investigated and compared in the mechanical case. The use of the higher order models was shown to decrease the error obtained between the numerical and analytical results which cannot be achieved through numerical refinement alone. The use of a higher order plate element type increases the rate of convergence to the numerical solution with less mesh elements, and decreasing the time step in the model reduces the obtained error. It was also found that $A_{0}$ wave propagation is more computationally expensive as more refined model kinematics and numerical mesh refinement is needed to achieve satisfactory results compared with $S_{0}$ wave propagation. A coupled electromechanical case was also proposed, where the Lamb waves were actuated and sensed using surface piezoelectric sensors and actuators. It was found that the used model was able to capture the electric potential signal generated due to wave propagation, which was then analysed to study the wave propagation and convergence using different model kinematics. The obtained results conformed with the mechanical case, where the error is reduced under higher order through-the-thickness expansion. A Node Dependent Kinematics (NDK) problem was also introduced in both cases, where the effect of reduction of the computational cost was studied with respect to the obtained error. It was shown that it is possible to use such models to decrease the computational cost of the proposed set-up up 40% while reducing the accuracy of the model by less than 1%. It was also shown that for $A_{0}$ problems, higher order kinematics need to be incorporated into an NDK model to achieve an acceptable accuracy compared to a lower order used in $S_{0}$ models. The nature of the reflected wave in the electro-mechanical model was investigated. It was shown that due to the asymmetry of the mounted sensor, the nature of the resulting reflection might change. This is especially applicable for $S_{0}$ travelling waves where the reflection seems to have an antisymmetric shape. This was not observed in symmetric double surface mounted sensors.

For further research, it is suggested to study the convergence of numerical models by implementing laminated structures, i.e., composite materials using Lamb wave propagation. It is also suggested to use a network of sensors and actuators, while exploiting the full potential of the Node Dependent Kinematics approach to substantially decrease the computational cost. This work would serve the purpose of accurate damage detection and structural health monitoring (SHM) in thin panels with a lower cost. Comparison of the damage positions obtained numerically under such models with those obtained experimentally is also of interest in the future, with or without taking into consideration the damping of the material and its effect on the attenuation of the propagating waves.

## Figures and Tables

**Table 1:** Material data of aluminium.

Youngs Modulus (E)	Poissons Ratio (ν)	Density (ρ)	Longitudinal Speed ($c_{1}$)	Transversal Speed ($c_{2}$)
$7\times 10^{10}\;{N/m}^{2}$	0.33	$2700\;{\mathrm{kg}/m}^{3}$	6197m/s	3121m/s

**Table 2:** Analytical phase and group velocities calculated at f=477.5 kHz and d=2 mm [32].

$c_{p_{S0}}≃5316$	$c_{p_{A0}}≃2298$
$c_{g_{S0}}≃5130$	$c_{g_{A0}}≃3126$

**Table 3:** Effect of using NDK approach in the mechanical case on accuracy and computational cost reduction.

		$S_{0}$		$A_{0}$		
NDK Model	DOF	Time (μs)	Error (%)	Time (μs)	Error (%)	${\mathrm{DOF}}_{\mathrm{red}}\;($%)
CLT	100,854	-	-	38.238	40.111	53.32
FSDT	100,854	-	-	60.462	5.303	53.32
TE1	100,854	38.404	1.523	60.462	5.303	53.32
TE2	129,654	38.874	0.318	60.961	4.522	39.99
TE3	158,454	38.996	0.005	63.417	0.675	26.66
TE4	187,254	38.998	0.000	63.839	0.014	13.33
TE5	216,054	38.998	0.000	63.848	0.000	0.00

**Table 4:** Structural, piezoelectric and dielectric properties of PZT-5A used in numerical model [37].

Property	$C_{11}$ (GPa)	$C_{12}$ (GPa)	$C_{13}$ (GPa)	$C_{33}$ (GPa)	$C_{44}$ (GPa)	$C_{66}$ (GPa)	$d_{31}$ (pm/V)	$d_{33}$ (pm/V)	$\epsilon_{33}^{T}/\epsilon_{0}$	$\epsilon_{33}^{S}/\epsilon_{0}$
PZT-5A	120	75.2	75.1	111	21	22.5	−190	390	1700	826

**Table 5:** Effect of using NDK approach in the electro-mechanical case on accuracy (error) and computational cost reduction.

		$S_{0}$		$A_{0}$		
NDK Model	DOF	Time (μs)	Error (%)	Time (μs)	Error (%)	${\mathrm{DOF}}_{\mathrm{red}}\;($%)
CLT	126,435	-	-	59.712	38.818	54.21
FSDT	126,435	-	-	92.004	5.732	54.21
TE1	126,435	58.335	1.762	91.966	5.771	54.21
TE2	172,548	59.184	0.332	93.472	4.228	37.51
TE3	218,580	59.378	0.005	96.820	0.797	20.84
LE 2B4	276,120	59.381	0.000	97.598	0.000	0.00

## Data Availability

The data required to reproduce these findings cannot be shared at this time as the data also forms part of an ongoing study.
